# Intracellular Trafficking and Translocation of Pertussis Toxin

**DOI:** 10.3390/toxins11080437

**Published:** 2019-07-25

**Authors:** Ken Teter

**Affiliations:** Burnett School of Biomedical Sciences, College of Medicine, University of Central Florida, Orlando, FL 32816, USA; kteter@mail.ucf.edu

**Keywords:** AB toxin, chaperone, endocytosis, endoplasmic reticulum, ERAD, retrograde transport, translocation

## Abstract

Pertussis toxin (PT) is a multimeric complex of six proteins. The PTS1 subunit is an ADP-ribosyltransferase that inactivates the alpha subunit of heterotrimeric G_i_/_o_ proteins. The remaining PT subunits form a pentamer that positions PTS1 in and above the central cavity of the triangular structure. Adhesion of this pentamer to glycoprotein or glycolipid conjugates on the surface of a target cell leads to endocytosis of the PT holotoxin. Vesicle carriers then deliver the holotoxin to the endoplasmic reticulum (ER) where PTS1 dissociates from the rest of the toxin, unfolds, and exploits the ER-associated degradation pathway for export to the cytosol. Refolding of the cytosolic toxin allows it to regain an active conformation for the disruption of cAMP-dependent signaling events. This review will consider the intracellular trafficking of PT and the order-disorder-order transitions of PTS1 that are essential for its cellular activity.

## 1. Introduction

*Bordetella pertussis* is the causative agent of whooping cough, a vaccine-preventable yet re-emerging infectious disease [1]. Approximately 150,000 global cases of whooping cough were reported in 2017 [2], with an increasing proportion of cases attributed to adolescents and adults rather than the historically affected population of young children [3,4]. A distinct paroxysmal cough that can last 8 weeks or longer is the hallmark symptom of this respiratory disease. Elevated lymphocyte counts in the bloodstream, hypoglycemia resulting from hyperinsulinemia, and possibly histamine sensitivity are additional complications of whooping cough [5]. Pertussis toxin (PT) plays a role in many of these complications, has additional immunomodulatory functions, and is essential for *B. pertussis* infection [6,7].

**Figure 1:**
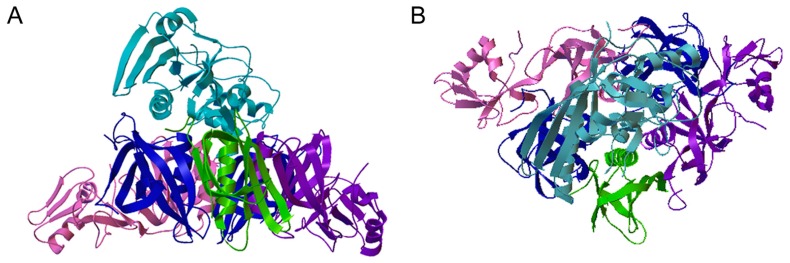
PT Ribbon Diagram. Side (**A**) and top-down (**B**) views of pertussis toxin (PT) are shown. PT has an AB structural organization, common to many toxins, that consists of an enzymatic A subunit and a cell-binding B subunit [8,9]. The A subunit (PTS1, light blue) sits above and within the central cavity of the B pentamer which is composed of an S2 subunit (purple), an S3 subunit (pink), two copies of the S4 subunit (blue), and an S5 subunit (green). PDB entry 1PRT [10].

PT is an AB-type protein toxin that contains a catalytic S1 subunit and a pentameric cell-binding subunit (Figure 1). Intoxication of a target cell involves transport of a stable PT holotoxin from the plasma membrane to the endoplasmic reticulum (ER), unfolding of the dissociated PTS1 subunit in the ER, PTS1 export to the cytosol through the ER-associated degradation (ERAD) pathway, and refolding of translocated PTS1 in the cytosol. The unfolding and refolding events are linked to the unstable nature of the free but not holotoxin-associated S1 subunit. This review will cover the cell biology of PT and the order-disorder-order transitions of PTS1 as it moves from the extracellular space, through the host endomembrane system, and into the host cytosol.

## 2. Order: The PT Holotoxin

### 2.1. Assembly and Stability of the PT Holotoxin

A single operon encodes the five proteins that comprise PT [11,12]. Each protein contains an N-terminal signal sequence that targets it to the periplasm, most likely through the general secretory pathway. After proteolytic removal of the signal sequence in the periplasm, the mature PT subunits assemble into an intact AB holotoxin that is pumped through the outer membrane and into the external environment by a type IV secretion system [13]. There are several intramolecular disulfide bonds within the individual PT subunits, but no intermolecular disulfide bond anchors the catalytic PTS1 subunit to the rest of the toxin [8,14]. PTS1 instead associates with the B pentamer through non-covalent interactions. Despite the lack of a covalent bridge between its A and B moieties, the PT holotoxin is remarkably stable—thermal denaturation occurs in a single event at 63 °C as recorded by differential scanning calorimetry [15].

### 2.2. PT Recognition of Target Cells and Receptor-Mediated Endocytosis

The PTB pentamer is built from a dimer of the S2 and S4 subunits, an S3/S4 dimer, and an S5 monomer that connects the two dimers. Despite the lack of intermolecular disulfide linkages, moderate levels of denaturant (5 M urea at pH 6.0) are required to release S5 from the dimers and more dramatic levels of denaturant (8 M urea at pH 8.4) are required to dissociate the two proteins in each heterodimer [8]. Furthermore, as determined by atomic force microscopy, thermal disassembly of the PTB pentamer only occurs after heating to 70 °C for 10 min [16]. The PTB pentamer is also stable over a pH range of 4.5 to 9.5 [16]. The S2 and S3 subunits both contain binding sites for sialic acid, a terminal carbohydrate found on many glycolipids and glycoproteins [17,18]. This likely explains the promiscuous adhesion of PT to a wide range of cell types and the lack of a single receptor for the toxin [19,20]. The variety of cells targeted by PT also explains its many physiological effects, which are reflected in the alternative names originally assigned to PT: lymphocytosis promoting factor, islet-activating protein, and histamine-sensitizing factor [21].

PT undergoes receptor-mediated endocytosis after binding to sialoconjugates on the surface of a target cell. Pinocytosis is not a functional internalization route for PT, as cells lacking sialylated glycoconjugates are resistant to the toxin [19,22,23]. Surface-bound PT has been visualized in clathrin-coated pits by electron microscopy, while intracellular PT is found in both coated and uncoated endocytic vesicles [24,25]. However, a functional role for clathrin-dependent endocytosis in PT intoxication has not been established. There are multiple mechanisms for receptor-mediated endocytosis [26], and the potential ability of PT to recognize many distinct sialylated surface receptors suggests it can use different receptors to access a range of endocytic mechanisms.

**Figure 2:**
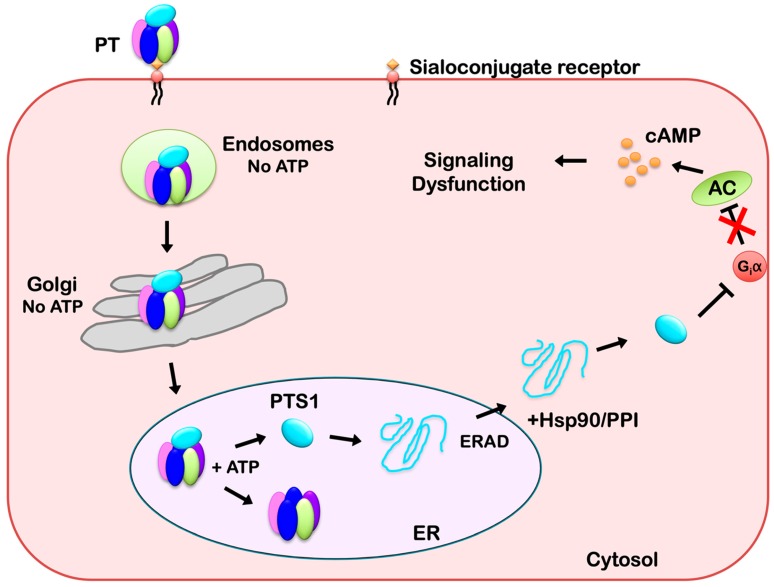
Intracellular Trafficking and Translocation of PT. PT binds to sialoconjugates on the surface of a target cell and is internalized by receptor-mediated endocytosis. It then moves as an intact holotoxin from the endosomes, through the Golgi apparatus, and to the endoplasmic reticulum (ER). The catalytic PTS1 subunit is held in a stable conformation by its association with the B pentamer, but it shifts to a disordered state when the ER-localized store of ATP triggers its release from the PT holotoxin. Unfolded PTS1 is subsequently recognized as ER-associated degradation (ERAD) substrate for export to the cytosol through a mechanism involving Hsp90 and a peptidyl-prolyl cis/trans isomerase (PPI). PTS1 refolds in the cytosol and inactivates Giα through ADP-ribosylation. Inactivated Giα can no longer down-regulate adenylate cyclase (AC), which leads to persistently elevated levels of intracellular cAMP and the disruption of signal transduction.

The functional pool of internalized PT moves from the endosomes, through the Golgi apparatus, and to the ER before PTS1 enters the cytosol to modify its G protein target (Figure 2). There are many distinct routes within this general pathway. For example, cargo can reach the Golgi apparatus from either an early endosomal compartment (i.e., the recycling endosomes) or from the late endosomes [27]. The specific transport route(s) followed by PT has not been elucidated, and only broad aspects of the endosomal trafficking pattern for PT are known—the pathway is pH-dependent and inhibited by the 22 °C temperature block that slows internalization from the plasma membrane and vesicle transport in the endosomal system [28,29,30,31]. The intracellular trafficking of PT is also microtubule-dependent [28], which could relate to both its movement within the endosomal system and its transport from the Golgi to the ER [32,33]. Likewise, the cholesterol-dependent route of PT trafficking [34] could involve both its endocytosis from the plasma membrane and its intra-Golgi transport [35,36]. Electron microscopy has visualized the accumulation of PT in multi-vesicular bodies that ferry cargo to the lysosomes and in the lysosomes themselves [24,25]. Thus, the majority of internalized PT appears to be delivered to the lysosomes for degradation. 

### 2.3. PT Transport from the Golgi Apparatus to the ER

A minor pool of internalized PT escapes delivery to the lysosomes and is instead shuttled to the Golgi apparatus and ER in a series of vesicle-mediated events collectively termed retrograde transport. There are multiple retrograde transport pathways [37], and the exact route(s) followed by PT is unknown. Given its promiscuous binding to sialoconjugates, it is possible that PT follows several endocytic and retrograde transport pathways. This has been demonstrated for ricin, an AB toxin that recognizes a common motif of terminal galactose residues on glycoproteins and glycolipids [38]. Each ricin receptor follows a distinct intracellular trafficking pattern, so the total cell-associated pool of ricin moves through several different transport pathways [39]. For both PT and ricin, most receptors deliver the toxin to the lysosomes for degradation. Only a subset of receptors will transport the surface-bound toxin to the Golgi and ER.

PT has been detected in the Golgi apparatus by subcellular fractionation [24], electron microscopy [25], immunofluorescence microscopy [25,34,40], and sulfation (a Golgi-specific modification) of recombinant S1 or S4 subunits that had been incorporated into a PT holotoxin [34]. Transport to the Golgi is required for intoxication, as cells with a temperature-sensitive Golgi apparatus are resistant to PT when grown at the restrictive temperature for Golgi function [24]. Brefeldin A, a fungal metabolite that dissolves the Golgi apparatus [41], also confers resistance to PT in a variety of cell lines [19,24,29,42]. These observations indicate transport to the Golgi apparatus is required for eventual PT delivery to the ER.

PT can be visualized in the endocytic pathway and Golgi apparatus, but it is not normally seen in the ER or the cytosol [34,42,43]. As determined by the highly sensitive method of surface plasmon resonance, only 3% of surface-associated PTS1 is delivered to the cytosol after 3 h of intoxication [42]. The low level of toxin that reaches the ER and eventually the cytosol is thus below the detection threshold for fluorescence microscopy. Transport to the ER has instead been documented using PT holotoxins with recombinant S1 or S4 subunits that were engineered to contain sites for N-linked glycosylation, a modification that begins in the ER and continues in the Golgi apparatus [34]. The sequential sulfation and then glycosylation of these recombinant toxins further demonstrated that PT reaches the Golgi apparatus before traveling to the ER [34]. 

PT must reach the ER in order to use a protein-conducting channel in the ER membrane for translocation to the cytosol. The ER contains two translocon systems (Hrd1 and Sec61) that can export proteins to the cytosol. However, both systems have channels that only accommodate individual proteins in linearized or partially unfolded states [44]. Thus, PTS1 must dissociate from the rest of the toxin before exiting the ER through a translocon pore.

## 3. Disorder: The Free PTS1 Subunit

### 3.1. PT Disassembly in the ER

AB toxin disassembly occurs before or concomitantly with A chain translocation to the cytosol. Disassembly usually requires the reduction of a disulfide bridge that anchors the catalytic subunit to the rest of the toxin, but there are no disulfide bonds between PTS1 and the PTB pentamer. PT disassembly involves an alternative mechanism in which ATP binds to the central pore of the B pentamer [45]. This alters the structure of the B pentamer and leads to the release of PTS1 from its holotoxin [46]. The intramolecular disulfide bond within PTS1 is subsequently reduced, which activates the latent enzymatic function of PTS1 [47,48,49]. Mutations that prevent ATP binding to the B pentamer thus prevent PT disassembly, PT activity against cultured cells, and *B. pertussis* colonization of the mouse respiratory tract [43]. 

ATP is not present in the periplasmic space where PT assembly occurs, and it is also absent from the endosomes and Golgi apparatus of the host cell. The only endomembrane organelle with a lumenal store of ATP is the ER [50,51]. Thus, PT will first encounter ATP when it reaches the ER translocation site. Hazes et al. [45] have noted that this allows ATP to serve as a molecular sensor for triggering holotoxin disassembly specifically at the site for PTS1 translocation to the cytosol.

The PT holotoxin and PTB pentamer are stable complexes [15,16], but the isolated PTS1 subunit has a highly disordered structure at the physiological temperature of 37 °C. As determined by near- and far-UV circular dichroism, the free PTS1 subunit has a tertiary structure transition temperature (T_m_) of 28.5 °C and a secondary structure T_m_ of 31 °C [52]. The ER-localized release of PTS1 from its holotoxin will therefore result in the spontaneous conversion of PTS1 to an unfolded state. This order-to-disorder transition has three functional consequences for the intoxication process: it (i) prevents holotoxin reassembly in the ER, (ii) identifies PTS1 as an ERAD substrate, and (iii) places PTS1 in a translocation-competent conformation for passage through the narrow diameter of a membrane-spanning translocon pore.

PTS1 can combine with the PTB pentamer at 4 °C or 7 °C to form a functional holotoxin [8,53], but toxin reassembly is unlikely to occur in the ER due to the disordered conformation of free PTS1 at physiological temperature. A similar 37 °C block of toxin reassembly has been documented for cholera toxin (CT), another ER-translocating AB toxin with a thermally unstable catalytic subunit [54]. For PTS1, reduction of its intrachain disulfide bond also prevents holotoxin reassembly at ambient temperature [53]. Two distinct mechanisms, thermal unfolding and disulfide reduction, thus prevent a futile cycle of ER-localized holotoxin disassembly and reassembly that could limit the pool of free PTS1 available for translocation to the cytosol. 

The release of PTS1 from its holotoxin leaves an intact PTB pentamer in the ER. At this point, PTB has fulfilled its two main functions: it has provided a stabilizing architecture for the otherwise heat-labile A chain, and it has delivered PTS1 to the ER translocation site. PTB does not play a direct role in the translocation event, as PTS1 can move from the ER to the cytosol when it alone is expressed in the ER from a plasmid-based transfection system [55,56]. PTB cycles between the ER and Golgi apparatus after the release of PTS1 [34], but it is not secreted back into the extracellular environment and does not enter the cytosol [42]. The PTB pentamer does not appear to play a functional role in the intoxication process after holotoxin disassembly, and its ultimate fate remains unknown. 

### 3.2. ERAD Processing of PTS1

Proteins enter the ER in a co-translational process that involves the passage of a linearized polypeptide through the Sec61 translocon pore. Folding begins as soon as the polypeptide chain appears at the lumenal face of the pore. This process is assisted by a network of chaperones, oxidoreductases, and lectins. The same factors also support protein assembly into multimeric complexes. Nascent proteins that fail to properly fold or assemble are recognized by the ERAD system, sequestered from the secretory pathway, and eventually exported through Hrd1 or Sec61 translocon pores for degradation in the cytosol [57]. ERAD thus provides PTS1 with a potential conduit to the cytosol. It was accordingly proposed that PTS1 masquerades as a misfolded protein for ERAD-directed export to the cytosol [58,59].

The hydrophobic C-terminus of PTS1 was originally thought to identify the toxin as an ERAD substrate [58]. To test this hypothesis, a plasmid-based system was used to directly express PTS1 or a C-terminal truncation of PTS1 in the ER of transfected cultured cells. After co-translational insertion into the ER via an N-terminal signal sequence, both constructs were exported to the cytosol where they ADP-ribosylated Giα. Removal of up to 54 amino acids from the C-terminus of PTS1 therefore had no effect on its export to the cytosol or its cytosolic activity [55,56]. 

Subsequent studies on the structure of the isolated PTS1 subunit provided an alternative mechanism to activate ERAD. These studies documented the intrinsic thermal instability of the free PTS1 subunit [52], which indicated PTS1 will unfold spontaneously when released from its holotoxin in the ER. Thus, PTS1 does not need to masquerade as a misfolded protein; it actually is an unfolded protein and a bona fide substrate for ERAD. The stabilization of PTS1 with chemical chaperones accordingly generates a toxin-resistant phenotype in cultured cells– if the dissociated PTS1 subunit cannot unfold in the ER, it cannot activate the ERAD system and will not reach the cytosol. This was demonstrated with intoxicated cells exposed to glycerol or 4-phenylbutyric acid [42]. Chemical chaperones that are used to treat protein folding disorders [60,61] could therefore be repurposed as a new class of toxin inhibitors.

The delivery of unfolded PTS1 to the lumenal face of a translocon pore is poorly characterized, and no specific ERAD factor that could guide this process has yet been identified. However, a functional role for ERAD in PTS1 translocation has been documented with the use of ERAD-defective cell lines. These cell lines export ERAD substrates to the cytosol at an attenuated rate [62], which limits the cytosolic accumulation of PTS1 and leads to a toxin-resistant phenotype [42]. As low levels of PTS1 could still be detected in the cytosol of the toxin-resistant cells, the quantity of cytosolic toxin appears to influence the extent of intoxication. 

Most exported ERAD substrates are appended with polyubiquitin chains that serve as a tag for proteasomal degradation [63]. Ubiquitin is conjugated to lysine residues on the target protein, but PTS1 exhibits an extreme arginine-over-lysine amino acid bias that eliminates the standard site for ubiquitin attachment: it has 22 arginine and 0 lysine residues [11,12,58,64]. This bias is not found in any of the PTB subunits, which indicates a strong evolutionary pressure to specifically eliminate lysine residues from the toxin A chain. The need to avoid ubiquitin-dependent proteasomal degradation again suggests that the quantity of cytosolic toxin is linked to the extent of intoxication. Worthington and Carbonetti [65] emphasized this point when they generated recombinant toxins with up to three arginine-to-lysine mutations in the PTS1 subunit. The in vitro activity of PTS1 was not affected by these mutations, but its in vivo activity was greatly reduced. The loss of cellular activity was apparently due to proteasomal degradation, as co-incubation with a proteasome inhibitor restored cellular activity to the recombinant toxins. Productive intoxication thus requires PTS1 to evade the usual ubiquitin-dependent proteasomal processing of an exported ERAD substrate.

### 3.3. PTS1 Extraction from the ER

The unfolding of the dissociated PTS1 subunit both activates the ERAD system and places the toxin in a translocation-competent conformation for passage into the cytosol. It is not known which translocon pore (Hrd1 or Sec61) facilitates the ER-to-cytosol export of PTS1, but passage through the pore will not occur spontaneously: a linearized polypeptide will oscillate back-and-forth through the membrane channel by Brownian motion, with no net movement in either direction. Cytosolic factors are therefore required to pull an unfolded protein through the membrane-spanning channel. This process provides the driving force for directionality to the translocation event. 

Hsp90, a cytosolic chaperone, is responsible for extracting the A chains of ADP-ribosylating toxins through a membrane pore [66,67]. For the catalytic A1 subunit of CT, toxin translocation is driven by the Hsp90-stimulated refolding of CTA1 [68]. A gain-of-structure at the cytosolic face of the ER membrane prevents CTA1 from moving back into the translocon pore, thus providing a Brownian ratchet for unidirectional ER-to-cytosol export [69]. The process begins when Hsp90 binds to an RPPDEI sequence spanning residues 11–16 of the CTA1 polypeptide [70]. PTS1 contains a similar N-terminal RPPEDV sequence (amino acids 13–18) that is recognized by Hsp90 [70], so it is likely that the binding of Hsp90 to this motif allows the chaperone to couple PTS1 refolding with PTS1 extraction from the ER. A functional role for Hsp90 in PTS1 translocation to the cytosol has been established [70], but the predicted refolding of disordered PTS1 by Hsp90 has not yet been documented.

Peptidyl-prolyl cis/trans isomerases (PPIs) often work with Hsp90 to facilitate toxin translocation from the endosomes to the cytosol [66,71]. PT intoxication requires PPI activity as well, as cells treated with a PPI inhibitor were resistant to PT, with an accumulation of toxin in the ER [72]. Interestingly, CTA1 translocation to the cytosol does not require PPI activity [73]. The molecular details of PTS1 export to the cytosol are thus distinct from CTA1 as well as other ER-translocating toxins such as ricin that do not utilize an Hsp90-dependent translocation mechanism [74,75]. 

## 4. Order: The Cytosolic PTS1 Subunit

### 4.1. The ADP-Ribosyltransferase Activity of PTS1

PTS1 uses NAD as the donor molecule for the ADP-ribosylation of cysteine residue 351 in the alpha subunit of G_i_/G_o_ [76]. The ADP-ribosylated form of Gα_i/o_ is locked in an inactive state that cannot down-regulate the function of adenylate cyclase. The intracellular concentration of cAMP consequently rises, leading to the dysfunction of host signaling pathways. Cellular processes affected by the PT-driven accumulation of cAMP include, depending on the affected cell type, histamine sensitization, insulin secretion and signaling, chemokine signaling, upregulation of proinflammatory cytokines, and suppression of adaptive immune responses [13,20,77]. Some of these outcomes, as well as other cellular effects resulting from PTS1 activity, may involve cAMP-independent signaling and/or signaling from the dissociated βγ subunits of the heterotrimeric G protein [5,78,79,80].

Reduction of the intramolecular disulfide bond between cysteine residues 41 and 201 in PTS1 is required for its ADP-ribosyltransferase activity [48,81]. Cysteine residue 41 is within the NAD binding pocket of PTS1 [13], and the alkylation of this residue inactivates PTS1 [53,82]. The ER-localized reduction of PTS1 thus plays two key roles in the intoxication process—it prevents reassociation of free PTS1 with the B pentamer and places PTS1 in a functional, NAD-binding conformation.

### 4.2. Refolding and Activation of Cytosolic PTS1

PTS1 will enter the cytosol in a linearized state as it passes through the translocon pore and must therefore regain an ordered, active conformation in the cytosol. This will not occur spontaneously due to the intrinsic instability of the free S1 subunit. In fact, a 2 min incubation at 42 °C is sufficient for the irreversible inactivation of free but not holotoxin-associated PTS1 [83]. Host factors must therefore return cytosolic PTS1 to a folded, functional conformation.

The activation of cytosolic PTS1 is poorly characterized but likely involves multiple interactions that refold and/or stabilize the translocated toxin. It is possible that Hsp90 remains associated with PTS1 after extracting the toxin from the ER. The chaperone-bound toxin would thereby remain in a folded conformation after entering the cytosol. This appears to occur for CTA1, which has no in vitro enzymatic activity at 37 °C in the absence of cellular factors such as Hsp90 [68,84,85]. Another host factor, ADP-ribosylation factor 6, can stabilize folded CTA1 but cannot induce a gain-of-function in the disordered toxin [86]. NAD may perform this stabilizing function for PTS1. In support of this possibility, NAD prevents the conversion of PTS1 to a protease-sensitive conformation when the toxin is heated to 37 °C [52] and helps reduced PTS1 retain its enzymatic activity at 30 °C [82]. PTS1 exhibits in vitro activity at 37 °C [83], but in these experiments, NAD was apparently added to PTS1 before its heating to 37 °C. NAD thus stabilizes the heat-labile PTS1 subunit and could possibly return disordered PTS1 to a functional conformation.

### 4.3. PTS1 Clearance from the Cytosol

PTS1 has no lysine residues for ubiquitin conjugation, and no other cellular modifications that could target PTS1 for proteasomal degradation have been identified. Yet, PTS1 is still a target for proteasomal degradation, as elevated levels of PTS1 were detected in the cytosol of cells co-incubated with PT and the proteasome inhibitor ALLN [42]. The intrinsic instability of the isolated PTS1 subunit likely accounts for its ubiquitin-independent proteasomal degradation. The proteasome is a large, barrel-shaped complex with three distinct proteolytic activities that all function on substrates within the barrel. Only unfolded proteins can move into the barrel of this core 20S proteasome. A 19S cap at one or both ends of the 20S proteasome recognizes ubiquitinated substrates and unfolds them for passage into the hollow cylinder of the proteasome. The combination of the 19S cap and 20S core forms a 26S proteasome that is responsible for the turnover of most cytosolic proteins. Without the 19S cap, the core 20S proteasome can only act on unfolded substrates [87]. The free PTS1 subunit represents one of these unfolded substrates, as we have reported that the purified 20S proteasome will degrade PTS1 at 37 °C [52]. Holotoxin-associated PTS1 and the PTB pentamer were not affected by the 20S proteasome, providing evidence that the disordered conformation of PTS1 renders it susceptible to degradation. Dissociation from the stabilizing host factor(s) that places cytosolic PTS1 in a functional conformation would thus lead to the ubiquitin-independent degradation of unfolded PTS1 by the core 20S proteasome. A slow rate of dissociation from its stabilizing host factors would ensure PTS1 persists in the cytosol long enough to modify its G protein target for a cytopathic effect. 

## 5. Conclusions

Several ER-translocating toxins contain unstable A chains that follow what Ampapathi et al. [88] termed order-disorder-order transitions during the intoxication process. PT follows this general pattern as well. The PT holotoxin is released as a stable complex into the external milieu through a type IV secretion system, enters a target cell through receptor-mediated endocytosis, and travels by retrograde transport to the ER. PTS1 is then released from the rest of the toxin and consequently shifts to a disordered state. This prevents the non-productive reassociation of PTS1 with the PT B pentamer and triggers the ERAD-mediated export of PTS1 to the cytosol. Most ERAD substrates are degraded by the ubiquitin-proteasome system, but PTS1 avoids this fate and instead interacts with host factors to regain an ordered conformation for the ADP-ribosylation of its G_i_/o protein target. However, the free PTS1 subunit will be in a disordered state that is susceptible to ubiquitin-independent degradation by the core 20S proteasome. The intrinsic instability of the free PTS1 subunit thus influences its translocation, degradation, and activity.
